# Optimal computation of all tandem repeats in a weighted sequence

**DOI:** 10.1186/s13015-014-0021-5

**Published:** 2014-08-16

**Authors:** Carl Barton, Costas S Iliopoulos, Solon P Pissis

**Affiliations:** 1 King’s College London, London, UK; 2 University of Western Australia, Crawley, Australia; 3 Curtin University, Bentley, Australia

**Keywords:** Tandem repeats, Weighted sequences, IUPAC notation

## Abstract

**Background:**

Tandem duplication, in the context of molecular biology, occurs as a result of mutational events in which an original segment of DNA is converted into a sequence of individual copies. More formally, a *repetition* or *tandem repeat* in a string of letters consists of exact concatenations of identical factors of the string. Biologists are interested in approximate tandem repeats and not necessarily only in exact tandem repeats. A *weighted sequence* is a string in which a set of letters may occur at each position with respective probabilities of occurrence. It naturally arises in many biological contexts and provides a method to realise the approximation among distinct adjacent occurrences of the same DNA segment.

**Results:**

Crochemore’s repetitions algorithm, also referred to as Crochemore’s partitioning algorithm, was introduced in 1981, and was the first optimal O(nlogn)-time algorithm to compute all repetitions in a string of length *n*. In this article, we present a novel variant of Crochemore’s partitioning algorithm for weighted sequences, which requires optimal O(nlogn) time, thus improving on the best known $O\;\left(n^{2}\right)$-time algorithm (Zhang et al., 2013) for computing *all* repetitions in a weighted sequence of length *n*.

## Background

A fundamental structural characteristic of a string of letters is its periodicity. Closely related to periodicity is the notion of *repetition*. Repetitions in strings are highly periodic factors, that is, two or more adjacent identical factors. For instance, the string TATATATA is a repetition in the string CTATAGTCTATAGT. Clearly a string may contain a quadratic number of repetitions. In 1981, it was shown by Crochemore that there could be O(nlogn)*maximal repetitions* in a string of length *n* and an O(nlogn)-time, thus optimal, algorithm was presented [1]. In 1999, Kolpakov and Kucherov presented an O(n)-time algorithm to compute the most compact representation of all repetitions known as *runs*[2].

Tandem duplication, in the context of molecular biology, occurs as a result of mutational events in which an original segment of DNA is converted into a sequence of individual copies. It usually results from replication slippage or from certain recombination events, such as unequal crossing-over or unequal sister chromatide exchange [3]. In this context, the result of a tandem duplication event is termed a *tandem repeat*. It appears in both eukaryotic [4] and prokaryotic [5] genomes.

Through time, individual copies within a tandem repeat may change by additional, uncoordinated, mutations, and so only approximate tandem copies may be present. The major bottleneck in identifying biologically relevant tandem repeats in genomic sequences is a certain variation threshold that must be admitted between the copies of the repeated segment. In other words, biologists are interested in *approximate* tandem repeats and not necessarily only in exact tandem repeats. A plethora of algorithms and tools for the identification of tandem repeats measuring this approximation have already been released (for instance, see [6]-[8]).

The simplest and perhaps most widely-used notion for measuring this approximation is the notion of Hamming distance [9]. Another way of measuring this approximation is using a probabilistic model of biological sequences. Single nucleotide polymorphisms, as well as errors introduced by wet-lab sequencing platforms during the process of DNA sequencing, can occur in some positions of a DNA sequence. In some cases, these uncertainties can be accurately modelled as a *don’t care* letter. However, in other cases they can be more subtly expressed, and, at each position of the sequence, a probability of occurrence can be assigned to each letter of the nucleotide alphabet; this process gives rise to a *weighted sequence*. For instance, consider a IUPAC-encoded [10] DNA sequence, where the ambiguity letter MM occurs at some position of the sequence, representing either base AA or base CC. This gives rise to a weighted DNA sequence, where at the corresponding position of the sequence, we can assign to AA and CC an occurrence probability of 0.5.

A great deal of research has been conducted for computing various types of regularities in weighted sequences (for instance, see [11]-[15]). The efficiency of the proposed algorithms relies on the assumption of a given constant, the *cumulative weight threshold*, defined as the minimal probability of occurrence of factors in the weighted sequence. Recently, the authors of [16] proposed an $O\left(n^{2}\right)$-time algorithm for computing all tandem repeats in a weighted sequence of length *n*.

### Our contribution

In this article, we present the first optimal algorithm for computing all tandem repeats in a weighted sequence. We improve on the time complexity of the best-known algorithm for computing all tandem repeats in a weighted sequence of length *n* from time $O\left(n^{2}\right)$ to an optimal O(nlogn). A preliminary version of this work appeared in [17].

## Preliminaries

In order to provide an overview of our results and algorithms, we begin with a few definitions, generally following [18].

An *alphabetΣ* is a finite non-empty set of size *σ*, whose elements are called *letters*. A *string* on an alphabet *Σ* is a finite, possibly empty, sequence of elements of *Σ*. The zero-letter sequence is called the *empty string*, and is denoted by *ε*. The *length* of a string *x* is defined as the length of the sequence associated with the string *x*, and is denoted by |*x*|. We denote by *x* [*i*], for all 0≤*i*<|*x*|, the letter at index *i* of *x*. Each index *i*, for all 0≤*i*<|*x*|, is a position in *x* when *x*≠*ε*. It follows that the *i*-th letter of *x* is the letter at position *i* in *x*.

The *concatenation* of two strings *x* and *y* is the string of the letters of *x* followed by the letters of *y*. It is denoted by *xy*. A string *x* is a *factor* of a string *y* if there exist two strings *u* and *v*, such that *y*=*uxv*. Consider the strings *x*,*y*,*u*, and *v*, such that *y*=*uxv*. If *u*=*ε*, then *x* is a *prefix* of *y*. If *v*=*ε*, then *x* is a *suffix* of *y*. Let *x* be a non-empty string and *y* be a string. We say that there exists an *occurrence* of *x* in *y*, or, more simply, that *xoccurs iny*, when *x* is a factor of *y*. Let *x* and *y* be two strings on *Σ*, such that |*y*|≥|*x*| and *x*=*y* [*i*..*j*]. We say that *x* occurs at the *starting positioni* in *y*.

A weighted string *x* on an alphabet *Σ* is a finite sequence of *n* sets. Every *x* [*i*], for all 0≤*i*<*n*, is a set of ordered pairs (*s*_*j*_,*π*_*i*_ (*s*_*j*_)), where *s*_*j*_∈*Σ* and *π*_*i*_ (*s*_*j*_) is the probability of having letter *s*_*j*_ at position *i*. Formally, $x\;\left[i\right]=\left\{\left(s_{j},\pi_{i}\;\left(s_{j}\right)\right)|s_{j}\neq s_{\ell }\;\text{for}\;j\neq \ell ,\;\text{and}\;\underset{j}{\sum }\pi_{i}\;\left(s_{j}\right)=1\right\}$. A letter *s*_*j*_ occurs at position *i* of a weighted string *x* if and only if the *occurrence probability* of letter *s*_*j*_ at position *i*, *π*_*i*_(*s*_*j*_), is greater than 0. A string *u* of length *m* is a factor of a weighted string if and only if it occurs at starting position *i* with *cumulative occurrence probability*$\overset{m-1}{\underset{j=0}{\prod }}\pi_{i+j}\left(u\left[j\right]\right)>0$. Given a *cumulative weight threshold* 1/*z*∈(0,1], we say that factor *u* is *valid*, or equivalently that factor *u* has a valid occurrence, if it occurs at starting position *i* and $\overset{m-1}{\underset{j=0}{\prod }}\pi_{i+j}\left(u\left[j\right]\right)\geq 1/z$. For clarity of presentation, in the rest of this article, a set of ordered pairs in a weighted string is denoted by [(*s*_0_,*π*_*i*_(*s*_0_)),…,(*s*_*σ*−1_,*π*_*i*_(*s*_*σ*−1_))].

### **Example1**.

Let the following weighted string *x* and the cumulative weight threshold 1/*z*=1/4.

TGTCATTGTCAT is not a factor of *x*; TATCCTTATCCT is a factor of *x* starting at position 3; and TATCATTATCAT is a valid factor of *x* starting at position 3 with cumulative occurrence probability 0.3.

For every string *x* and every natural number *n*, we define the *n*-th power of the string *x*, denoted by *x*^*n*^, by *x*^0^=*ε* and *x*^*k*^=*x*^*k*−1^*x*, for all 1≤*k*≤*n*. A string is said to be *primitive* if it cannot be written as *v*^*e*^, where *e*≥2. A repetition in *x* is a non-trivial power of a primitive string occurring in *x*.

Formally, a *repetitionu*^*e*^, *e*≥2, in *x* is defined as a triple (*i*,*p*,*e*) such that: *u*=*x*[*i*..*i*+*p*−1]=*x* [*i*+*p*..*i*+2*p*−1]=…=*x* [*i*+(*e*−1)*p*..*i*+*ep*−1]; *u*^*e*+1^ does not occur at position *i*; and *u* is primitive. A repetition is maximal if *i*−*p*<0 or *u*^*e*^ does not occur at *x* [*i*−*p*]. The integers *p* and *e* are called the *period* and the *exponent* of the repetition, respectively. In other words, a repetition is a primitively-rooted integer power *u*^*e*^ which is not followed by another occurrence of *u*; and a maximal repetition is a primitively-rooted integer power *u*^*e*^ which is not followed or preceded by another occurrence of *u*. If *e*=2 the repetition is called *square*.

A *repetitionv*=*u*^*e*^, *e*≥2, in a weighted string *x* is defined as a quadruple (*i*,*p*,*b*,*e*) such that *u*=*v* [0..*p*−1]=*v* [*p*..2*p*−1]=…=*v*[(*e*−1)*p*..*ep*−1], where *v* is a factor of length *ep* of *x* occurring at position *i*, and each occurrence of *u* in *v* is a valid factor of *x*; *u*^*e*+1^ does not occur at position *i*; *u* is primitive; and *b* is a set of ordered pairs (*j*,*a*), where 0≤*j*<*p* and *a*∈*Σ*, denoting *u* [*j*]=*a*. A repetition is maximal if *i*−*p*<0 or *u*^*e*^ does not occur at *x* [*i*−*p*]. The need for set *b* in uniquely defining a repetition can be seen in Example 2.

### **Example2**.

Let *x*= aab [(a,0.5)(b,0.5)] [(a,0.5)(b,0.5)]bab and 1/*z*=1/2. Then (1,3 {(2,a)},2) is a repetition in *x*, such that *u*=aba and *v*=abaaba.

In this article, we are mainly concerned with the following problem.

### **Problem1**.

Given a weighted string *x* of length *n* and a cumulative weight threshold 1/*z*∈(0,1], find all repetitions in *x*.

## Algorithm

In the following discussion, we assume that each position contains at least one letter with occurrence probability greater than or equal to 1/*z*. If this is not the case, the weighted string can be split around these positions, and each resulting weighted string can be processed separately according to the algorithm with no time penalty.

The first stage of the algorithm is to perform a simple filtering on the weighted string to filter out all those letters that are below the threshold. This is required as if the alphabet is not constant, we may have many letters with low occurrence probability that are not of interest. We simply read the entire string and keep only those letters with occurrence probability greater than or equal to 1/*z*; these are at most *z* for each position, so still constant. We are thus left with a string of size O(n), and the entire stage takes time O(σn). For clarity of presentation, in the rest of this article, we assume that the string resulting from this filtering step is the input weighted string *x*.

After this filtering stage, we perform a colouring stage on *x*, similar to the one before the construction of the weighted suffix tree [12], which assigns a colour to every position in *x* according to the following scheme:

 mark position *iblack* (BB), if *none* of the possible letters at position *i* has occurrence probability greater than 1−1/*z*.

 mark position *igrey* (GG), if *one* of the possible letters at position *i* has occurrence probability greater than 1−1/*z*.

 mark position *iwhite*(WW), if *one* of the possible letters at position *i* has occurrence probability of occurrence 1.

It should be noted that the colouring stage only applies when *z*≥2; should it be less than 2, then all positions are either grey or white. An example of the colouring stage can be seen in Table 1. Intuitively, black positions are the only positions where multiple letters may be chosen for any valid factor that includes the black position. Due to this, black positions are also called *branching positions*.

**Table 1:** **Colouring of** ***x* for 1/*z*** **=1/2**

**Position**	**0**	**1**	**2**	**3**	**4**	**5**	**6**	**7**	**8**	**9**	**10**
*x*	AA	(AA, 0.1)	TT	TT	(AA, 0.5)	TT	CC	(AA, 0.6)	TT	TT	TT
		(CC, 0.8)			(CC, 0.5)			(CC, 0.2)			
		(GG, 0.1)			(GG, 0.0)			(GG, 0.0)			
		(TT, 0.0)			(TT, 0.0)			(TT, 0.2)			
**Colour**	WW	GG	WW	WW	BB	WW	WW	GG	WW	WW	WW

After the colouring stage, we perform a generation stage, similar to the one performed during the construction of the weighted suffix tree, where a set of factors of *x* is generated; we refer to this set as *extended factors*. The intuition behind extended factors is to generate a set of strings such that all the valid factors of the weighted string occur in at least one extended factor. The generation of extended factors is performed once from each black position. We scan *x* from left to right and for the currently considered black position, we create a list of possible extended factors starting from this position. We generate a factor starting with each letter at the black position and one empty string. These will then be extended to create the extended factors starting from that black position. For each extended factor, the cumulative occurrence probability is maintained during its generation, and when it breaks the threshold we stop extending it. This probability is updated by considering the actual occurrence probability for letters at black positions, but letters at grey positions are treated as letters at white positions (only one possible choice). Extending these factors is performed by continuing to scan *x* and appending to the currently considered factors the same single letter if the position is white or grey and by creating new factors at black positions. At a black position, we copy each current extended factor and append one letter from the black position to each copy. We stop extending an extended factor when we reach a black position which causes it to violate the threshold. The procedure outlined above is similar to the generation step in [12], however, here, we generalise the procedure to any finite alphabet, by noting that the branching factor for any finite alphabet can be no more than 1/(1/*z*)+1=*z*+1 for the first branching and *z* for the rest; as no more than *z* letters can have an occurrence probability greater than or equal to 1/*z*.

### **Example3**.

Let *x*= aab [(a,0.5)(b,0.5)] [(a,0.5)(b,0.5)]bab and 1/*z*=1/2. The colouring corresponding to *x* is WWWBBWWW. We scan *x* from left to right and extend until the first black position. At this point we have factor aab and at the black position we branch and get aaba and aabb. Extending these any further will violate the threshold so we stop. The extended factors generated from position 0 of *x* are as follows: aaba and aabb. We continue with factors a, b, and *ε* at the black position 3. We then reach the black position 4 and for factors a and b this violates the threshold so we stop. However, the empty string can be extended, so we split it into two strings, as we are at a black position, and continue extending it. The extended factors generated from the black position 3 are as follows: a, b, abab, and bbab. We continue with factors a, b, and *ε* at the black position 4. The extended factors generated from the black position 4 are as follows: abab, bbab, and bab.

We recall an important lemma on how many black positions may be contained within any valid (or extended) factor of a weighted string. We also give a slightly modified proof for any finite alphabet.

### **Lemma1** ([12]).

Given a weighted string *x* and a cumulative weight threshold 1/*z*∈(0,1], any valid factor of *x* contains at most $\left\lceil \log z/\log\;\left(\frac{z}{z-1}\right)\right\rceil$ black positions.

### *Proof*.

Consider a valid factor *u* of *x* containing *ℓ* black positions and no grey positions. Any letter at a black position has occurrence probability at most 1−1/*z*. The cumulative occurrence probability of *u* with *ℓ* black positions is no more than (1−1/*z*)^*ℓ*^, and it must be the case that (1−1/*z*)^*ℓ*^≥1/*z* since *u* is valid; by rearranging and taking logarithms we obtain the claimed result.

Note that, additionally, Lemma 1 holds exactly for extended factors as they are factors which are treated as only containing black and white positions. From the generation of extended factors we get the following.

### **Lemma2** ([12]).

Given a weighted string *x* and a cumulative weight threshold 1/*z*∈(0,1], any valid factor of *x* occurs in at least one extended factor.

To achieve the main result of the article, we first solve a related sub-problem on the computation of *valid repetitions*. Additionally, we define the notion of an *extended repetition* in the process. An *extended repetition* in *x* is a repetition occurring in an extended factor of *x*. A *valid repetition* in *x* is a repetition *u*^*e*^ such that the cumulative occurrence probability of *u*^*e*^ is at least 1/*z*. We are now in a position to define the following subproblem.

### **Problem2**.

Given a weighted string *x* of length *n* and a cumulative weight threshold 1/*z*∈(0,1], find all valid repetitions in *x*.

Intuitively, the difference between Problems 1 and 2 is that in Problem 1 we want to find repetitions *u*^*e*^ such that *eachu* occurs with cumulative occurrence probability at least 1/*z*; whereas in Problem 2 we want to find repetitions *u*^*e*^ such that the *entire* factor *u*^*e*^ occurs with cumulative occurrence probability at least 1/*z*.

### **Lemma3**.

Given a weighted string *x* and a cumulative weight threshold 1/*z*∈(0,1], any valid repetition in *x* occurs in at least one extended factor.

### *Proof*.

By Lemma 2, any valid factor of *x* occurs in at least one extended factor. By the definition of valid repetitions, any valid repetition is a valid factor.

For each generated extended factor, we run Crochemore’s partitioning algorithm for maximal repetitions; the output is all maximal extended repetitions in *x*. After computing all maximal extended repetitions, we cannot simply report all of these as valid repetitions. All valid factors must occur in an extended factor but extended factors may contain factors which are not valid. This is a consequence of treating grey positions as white during the generation of extended factors [12]. Since not all maximal extended repetitions are valid repetitions, we must therefore break up these maximal extended repetitions into valid repetitions to solve Problem 2.

In order to break up the maximal extended repetitions, we must compute some additional information. To determine how long any valid repetition should be, we must know, for each position *i* in an extended factor, the length of the longest valid factor starting at position *i*. The computation is based on the observation that the longest factor with cumulative occurrence probability greater than or equal to 1/*z* for the position *i*+1 has length greater than or equal to that of position *i*. To compute this we maintain an additional cumulative weight threshold *π*^′^. This additional threshold is reused so that we can easily compute the longest valid factor for some position *i*+1 from position *i*. We store the computed lengths in an array LF of integers.

We start with the first position in an extended factor and compute the longest factor within the threshold by multiplying together the occurrence probability of the letters we encounter and storing this in *π*^′^. If multiplying the probability of some letter at position *j*>0 causes *π*^′^<1/*z*, we set LF [0]:=*j*−1. To proceed, we remove by division the occurrence probability of the first letter from *π*^′^. If *π*^′^<1/*z*, then we set LF [1]:=*j*−1; otherwise, we continue as before multiplying the occurrence probability of letters at positions *j*+1,*j*+2, and so on, until the threshold is once again violated. In general, for string *x* and some position *i*, we set $\text{LF}\;\left[i\right]:=\max\;\left\{|u|:\overset{|u|-1}{\underset{j=0}{\prod }}\pi_{i+j}\left(u\;\left[j\right]\right)\geq 1/z\right\}$, where non-empty factor *u* occurs at starting position *i* of *x*.

### **Example4**.

Let *x*= [(a,0.6)(c,0.4)] bab [(a,0.6)(d,0.4)]bab and 1/*z*=1/2. The colouring corresponding to *x* is GWWWGWWW. The only extended factor generated by this weighted string is abababab. Starting at position 0, we set *π*^′^:=0.6, that is, the probability that a occurs at position 0. We then consider the cumulative occurrence probability of factors ab, aba, and abab, the probability of whose is also 0.6. Now we consider the next letter at position 4 and set *π*^′^:=0.36<1/2. We set LF [0]:=4, and remove the occurrence probability of the first letter from the cumulative occurrence probability by setting *π*^′^:=0.36/0.6=0.6. Now we continue as before and consider the following factors (which now start from position 1) baba, babab, bababa, and bababab. We reach the end of the string without breaking the threshold, and so we set LF [1]:=7, LF [2]:=6, LF [3]:=5, and so on until LF [*n*−1]:=1.

For each extended factor this takes time and space proportional to its length. The sum of lengths of the extended factors is linear in *n* by Lemma 5. We give an alternative proof to the one given in [12] for any finite alphabet, both for completeness and as we improve the bounds on the constants slightly. For this alternative proof, we first need to show the following lemma.

### **Lemma4**.

Given a weighted string *x* and a cumulative weight threshold 1/*z*∈(0,1], any valid factor of *x* occurs in at most *z*^*ℓ*^(2*ℓ*+1) extended factors of *x*, where $\ell =\left\lceil \log z/\log\;\left(\frac{z}{z-1}\right)\right\rceil$.

### *Proof*.

By the definition of extended factors, each black position initially generates *z*+1 extended factors; *z* including the current black position and one that does not. At each subsequent black position, each extended factor may branch at most *z* times, and this occurs no more than *ℓ* times. From this we get that a black position generates no more than *z*^*ℓ*^+*z*^*ℓ*^ extended factors; *z*^*ℓ*^ from those that initially include the current black position and another *z*^*ℓ*^ from the one that does not.

Now consider some position *i* in the weighted string *x*. Position *i* can only be in extended factors generated by black positions to the left of it or at position *i* itself; and we know that an extended factor can contain at most *ℓ* black positions. Position *i* can only be contained in extended factors generated from the *ℓ*+1 black positions to the left. For the first *ℓ* to the left, it can be contained in any extended factor but for the *ℓ*+1th black position to the left, it can only be contained in those extended factors which do not include the *ℓ*+1th black position.

By Lemma 2, all valid factors occur in extended factors and no valid factor can occur in strictly more extended factors than its respective single-letter valid factors. Therefore it is sufficient to determine, for some position *i*, the maximum number of occurrences of its single-letter valid factors in extended factors. From the abve analysis, we can see that each position can be in at most *ℓz*^*ℓ*^+*z*^*ℓ*^=*z*^*ℓ*^(2*ℓ*+1) extended factors.

We are now ready to establish the sum of lengths of extended factors.

### **Lemma5** ([12]).

Given a weighted string *x* of length *n* and a cumulative weight threshold 1/*z*∈(0,1], the sum of lengths of the extended factors of *x* is O(n).

### *Proof*.

Following the proof of Lemma 4, we see that each position is in no more than *z*^*ℓ*^(2*ℓ*+1) extended factors. To establish the sum of lengths of extended factors it is sufficient to count how many extended factors each position is in; therefore the sum of lengths of all extended factors is no more than $z^{\ell }(2\ell +1)n=O(n)$.

The next step is to determine the set *b* for each maximal extended repetition. This can be done in constant time per maximal extended repetition. We compute an array NB of integers of size *n*, such that for each position *i* in *x*, NB[*i*] stores the index of the leftmost black position *j*>*i*; this can be done in linear time in *n*. For each maximal extended repetition *u*^*e*^, we check all black positions in the first occurrence of *u*. By Lemma 1, there can only be a constant number of black positions in *u*; finding the black positions using NB takes time proportional to their number. It is now a simple case of recording the position and the letter present in the extended factor; this takes constant time per maximal extended repetition, so time proportional to the number of maximal extended repetitions in total.

Given all the maximal extended repetitions, we can now begin to break them up into valid repetitions. To achieve this, we can check the length of the longest valid factor starting at position *i* of the extended factor, and then determine the longest valid repetition starting from *i*. We can continue checking the maximal extended repetition in this manner reporting the length as we go. Note that in the worst case, for each maximal extended repetition *u*^*e*^, we may check the starting position of each occurrence of *u*. As we show later (Lemma 7), this can be done efficiently. We now establish the maximal number of extended repetitions in *x*. Note that the work done by the algorithm so far is no more than the maximal number of extended repetitions.

### **Lemma6**.

Given a weighted string *x* of length *n* and a cumulative weight threshold 1/*z*∈(0,1], there could be O(nlogn) extended repetitions in *x*.

### *Proof*.

Consider the string *x* partitioned into *q* non-overlapping segments *N*_*i*_, 1≤*i*≤*q*, each of which contains $\ell =\left\lceil \log z/\log\;\left(\frac{z}{z-1}\right)\right\rceil$ black positions. Each segment starts with the first black position of the segment and ends immediately before what would otherwise be the *ℓ*+1th black position of the segment. For some segment *N*_*i*_, each black position may generate 2*z*^*ℓ*^ extended factors. By the definition of extended factors, each extended factor may contain no more than *ℓ* black positions; so none of the extended factors can extend past the next segment *N*_*i*+1_. Each of these extended factors may contribute to the number of extended repetitions. There may be no more than 2*ℓz*^*ℓ*^ extended factors from any *N*_*i*_, and each is of length no more than |*N*_*i*_|+|*N*_*i*+1_|; so each extended factor may contribute $O\;\left(\left(\left|N_{i}|+|N_{i+1}\right|\right)\log\;\left(\left|N_{i}|+|N_{i+1}\right|\right)\right)$ extended repetitions. Each segment may contribute no more than $O\;\left(2\ell z^{\ell }\left(\left|N_{i}|+|N_{i+1}\right|\right)\log\;\left(\left|N_{i}|+|N_{i+1}\right|\right)\right)=O\;\left(\left(\left|N_{i}|+|N_{i+1}\right|\right)\log\;\left(\left|N_{i}|+|N_{i+1}\right|\right)\right)$ extended repetitions. Summing the extended repetitions that each segment may contribute, we achieve our claim that the number of extended repetitions is O(nlogn).

As previously mentioned, whilst breaking some maximal extended repetition *u*^*e*^ into valid repetitions, we may need to check up to *e* positions. The maximum number of checks required will be the sum of the exponents of all maximal extended repetitions returned by Crochemore’s partitioning algorithm. Now we establish the maximal sum of the exponents of maximal extended repetitions in a weighted string.

### **Lemma7**.

Given a weighted string *x* of length *n* and a cumulative weight threshold 1/*z*∈(0,1], the sum of exponents of maximal extended repetitions in *x* is O(nlogn).

### *Proof*.

Any primitive repetition *u*^*e*^ can also be seen as a sequence of overlapping primitive squares (as shown in Example 6). We know that the maximal number of occurrences of primitive squares is O(nlogn)[19]; clearly the sum of the exponents of primitive squares is also O(nlogn). By the definition of maximal extended repetitions each square is only in one maximal extended repetition. Therefore the sum of exponents of maximal extended repetitions is less than or equal to the sum of exponents of primitive squares. This is also O(nlogn).

Note that an analogous version of Lemma 6 holds for valid repetitions. We are now in a position to state our first result.

### **Theorem1**.

Problem 2 can be solved in optimal time O(nlogn).

### *Proof*.

Consider the string *x* partitioned into *q* non-overlapping segments *N*_*i*_, 1≤*i*≤*q*, each of which contains $\ell =\left\lceil \log z/\log\;\left(\frac{z}{z-1}\right)\right\rceil$ black positions. The proof can follow, almost identically, that of Lemma 6 but instead of considering extended repetitions, we consider the time contributed by each segment; this too is $O\;\left(\left(\left|N_{i}|+|N_{i+1}\right|\right)\log\;\left(\left|N_{i}|+|N_{i+1}\right|\right)\right)$ per segment.

At this point, we have solved the subproblem which forms the basis for our solution. Intuitively, the subproblem finds repetitions *v*=*u*^*e*^, where factor *v* occurs with cumulative occurrence probability greater than or equal to 1/*z*. The idea behind our solution to Problem 1 is based on the observation that a repetition of exponent *e*≥3 is composed of overlapping occurrences of smaller repetitions. We intend to compute smaller repetitions and, from this, derive larger ones. Part of the process of computing valid repetitions was to break up maximal extended repetitions below the threshold into smaller valid repetitions. To determine the repetitions specified in Problem 1, we reverse this process and *compose* longer repetitions from small valid repetitions.

In order to solve Problem 1, we start by solving Problem 2 for threshold *k*=1/*z*^2^. The number of valid repetitions reported for *k* can be shown to be O(nlogn) by the same argument as for Lemma 6; and the number of black positions in a valid factor is only a constant amount higher than for the original threshold by a similar argument to the proof of Lemma 1. We pick *k*=1/*z*^2^ as we wish to guarantee that we will at least find squares such that each half may have cumulative occurrence probability greater than or equal to 1/*z*. We may also find repetitions with a higher exponent and repetitions which have a cumulative occurrence probability less than 1/*z*, but we will explain how to filter these out using the same techniques as for Problem 2.

We alter the solution to Problem 2 to simplify the solution to Problem 1. Instead of breaking up maximal extended repetitions into valid repetitions, we break them into all their valid overlapping squares. There are no more than O(nlogn) valid squares [19]. This can be shown by an almost identical argument as Lemma 6. To split maximal extended repetitions into their valid overlapping squares, we process them one by one and create a new square for each overlapping square in the maximal extended repetition. We only need to perform this on maximal extended repetitions of exponent *e*≥3, and this will take time proportional to the sum of the exponents which, by Lemma 7, is O(nlogn).

To perform the filtering step, we must check if both halves of the square are above the threshold 1/*z*. To check each half, we compute, for each position *i* in an extended factor, the length of the longest valid factor starting at position *i*. During the generation of extended factors for the threshold *k*, we at the same time determine the longest factor with cumulative occurrence probability greater than or equal to 1/*z* by computing an array LF^′^ which stores the analogous information. Filtering the squares in time proportional to their number can be done by checking that the length stored in the array is greater than or equal to the period of the square.

After the filtering step, we have a set of quadruples (*i*,*p*,*b*,*e*) representing all primitive squares such that each half of a square has cumulative occurrence probability at least 1/*z*. Now, for every position *i* in *x*, we declare an array A_*i*_ of linked lists, such that the linked list A_*i*_[ *f*_*i*_(*j*)], $f_{i}:\left[1,\lfloor n/2\rfloor \right]\to \left[0,O\;\left(\underset{\phi }{\log}n\right)\right]$, stores all the squares which occur at position *i* with period *j*∈[1,⌊*n*/2⌋]. We now wish to establish the size of A_*i*_ and the size of the linked lists stored at any A_*i*_ [ *f*_*i*_(*j*)].

### **Lemma8**.

A_*i*_ is of size $O\;\left(\underset{\phi }{\log}n\right)$, where $\phi =\left(1+\sqrt{5}\right)/2$, and the size of any linked list A_*i*_[ *f*_*i*_(*j*)] is O(1).

### *Proof*.

There is a constant number of valid factors starting from position *i*, and it is well known that a string can contain no more than log*ϕn* prefixes that are squares [19]. By Lemma 4, position *i* is only in O(1) extended factors. The suffixes starting from *i* in each extended factor contain no more than log*ϕn* prefixes that are squares; this achieves the first part of our claim. For the second part, it is enough to note that each suffix of an extended factor starting from *i*, which there is O(1) of, can only contain one square of a given period.

We can now construct the repetitions specified in Problem 1. For each position *i*, we iterate through the linked lists of array A_*i*_. We iterate through each linked list A_*i*_ [ *f*_*i*_(*p*)], where *p* is the considered period. We process each square element (*i*,*p*,*b*,*e*)∈A_*i*_ [ *f*_*i*_(*p*)] to extend the corresponding square as much as possible, by checking for an occurrence of the square at position *i*+*p*. For a linear string, it is simple to determine this. For each pair of overlapping squares, the second half of the first square is the first half of the second square; so it suffices to check whether there exists a square at position *i*+*p* with the same period.

### **Example5**.

Consider *y*=ababab that contains the following primitive squares: (0,2,2), (1,2,2), and (2,2,2); we wish to find the repetition (0,2,3). We start at position 0 of *y* with (0,2,2) and check if there is a square of period 2 starting at position 2. A matching square exists so we extend the repetition and check position 4. There is no square at position 4 so we report the repetition (0,2,3).

For weighted strings the approach is very similar, with the addition of a few, constant-time, checks. We must check, for each pair of overlapping squares, if the black positions from the first square match with the black positions from the second square. There is a constant number of black positions so this takes constant time. Each time we find such overlapping squares, we extend our repetition and delete the square at position *i*+*p* from the corresponding list. As soon as we find a position where we cannot extend the repetition we stop. We continue doing this until we have found all repetitions.

### **Example6**.

Let *x*=aab[(a,0.5)(b,0.5)][(a,0.5)(b,0.5)]ab and 1/*z*=1/4. We would like to report repetition *v*=ababab, defined by (1,2 {*∅*},3). For *i*=1, we iterate through the linked lists of array A_1_. For *p*=2, we iterate through the linked list A_1_ [ *f*_1_(2)]. We find the square abab, defined by (1,2 {*∅*},3). We check for an occurrence of the same square at position *i*+*p*=1+2=3, and find (3,2,{(0,*a*),(1,*b*)},2) in A_3_ [ *f*_3_(2)]. We have to check if the black positions from the first square match with the black positions from the second square. They do, so we extend our square to repetition ababab, defined by (1,2 {*∅*},3), and delete the square at position *i*+*p*=3 from the list A_3_ [ *f*_3_(2)].

Each time we iterate through a linked list, a square may be added to the repetition we are extending; this takes constant time per list by Lemma 8. After each square is added to the repetition, it is deleted so is not considered again. There are O(nlogn) squares in the array and from the above description we can see that each square is considered a constant number of times. It is clear that we construct no more repetitions than there are primitive squares, so the number of constructed repetitions is also O(nlogn). These repetitions will be maximal, and to report repetitions specified in Problem 1, we may check the start of each occurrence in the repetition and report them. This takes no more than the sum of exponents which is O(nlogn). We can now state the main result of this article.

### **Theorem2**.

Problem 1 can be solved in optimal time O(nlogn).

## Conclusions

In this article, we presented an optimal algorithm for computing all tandem repeats in weighted sequences. We improved on the time complexity of the best-known algorithm for computing all tandem repeats in weighted sequences from $O\;\left(n^{2}\right)$ to an optimal O(nlogn), and showed that Crochemore’s partitioning scheme can be used efficiently in weighted sequences.

A most obvious drawback of the proposed algorithm is the hidden constant due to the generation of extended factors. This constant appears throughout the work. The high constant factor in the other operations is due to them being performed on extended factors—not due to an inherently large constant in the operation itself. It should also be noted that the existing algorithms for the computation of repetitions in weighted strings also suffer from this problem. All the algorithms make the assumption that the cumulative weight threshold is constant and generate the valid factors in one way or another, pruning out those that violate the threshold to avoid a combinatorial explosion. This exponential dependency is explicitly mentioned in [20], where an exponential constant is derived, and also in [16]. The difference between our approach and that of [16] is that we also improve the complexity from $O\;\left(n^{2}\right)$ to the optimal O(nlogn).

For future work, we intend on devising an algorithm to compute a most compact representation of all maximal repetitions in weighted strings similar to the one for regular strings [2]. It seems as though the techniques we have developed here are not directly applicable to this computation. Perhaps new methods will need to be developed to achieve a linear-time computation.

## Competing interests

The authors declare that they have no competing interests.

## Authors’ contributions

CB, CSI, and SPP devised the algorithms. CB and SPP wrote the manuscript. The final version of the manuscript is approved by all authors.
